# Empagliflozin protects diabetic pancreatic tissue from damage by inhibiting the activation of the NLRP3/caspase-1/GSDMD pathway in pancreatic β cells: in vitro and in vivo studies

**DOI:** 10.1080/21655979.2021.2001240

**Published:** 2021-11-25

**Authors:** Pan Liu, Zhengdong Zhang, Jinwu Wang, Xiao Zhang, Xiaoping Yu, Yao Li

**Affiliations:** a School of Clinical Medicine, Chengdu Medical College, Chengdu, China; b Department of Endocrinology, The First Affiliated Hospital of Chengdu Medical College, Chengdu, China; c Department of Orthopedics, The First Affiliated Hospital of Chengdu Medical College, Chengdu, China; d School of Biosciences and Technology, Chengdu Medical College, Chengdu, China; e School of Public Health, Chengdu Medical College, Chengdu, China

**Keywords:** Empagliflozin, pancreatic β cells, NLRP3, caspase-1, GSDMD

## Abstract

Diabetes mellitus is an important public health problem worldwide. Insulin deficiency caused by pancreatic β cell dysfunction is an important pathogenic factor of diabetes mellitus. This study evaluated whether empagliflozin (EMPA) protects the pancreas from diabetes mellitus-induced injury by downregulating the nucleotide-binding oligomerization domain-like receptor protein 3 (NLRP3)/caspase-1/Gasdermin D (GSDMD) pyroptosis-related inflammasome pathway *in vitro* and *in vivo. In vivo*, animals were separated into blank control (control, C57/bl6j wild-type mice), diabetes model (db/db mice, BKS-Lepr^em2Cd479^/Gpt mice), and db/db mice+EMPA (db/db+EMPA) groups. *In vitro*, pancreatic β cells were separated into low glucose (control), high glucose (HG), and HG+EMPA groups. The db/db+EMPA group were administered empagliflozin at 10 mg/(kg·day) by gavage for six months. Histological changes in the pancreatic tissues were observed by hematoxylin-eosin staining, and levels of the pyroptosis-related inflammatory factors NLPR3, caspase-1, and GSDMD were measured by immunohistochemistry and immunofluorescence staining methods. The Cell Counting Kit-8 assay was used to detect the effect of different concentrations of glucose and empagliflozin on the proliferation of mouse insulinoma islet β (β TC-6) cells. NLRP3/caspase-1/GSDMD expression was assessed by *western blotting* and immunofluorescent labeling in the β TC-6 cells. The results showed that empagliflozin reduced the pathological changes and inflammatory cell infiltration in the pancreatic tissues of db/db mice. Furthermore, empagliflozin not only reduced the expression levels of NLRP3/caspase-1/GSDMD *in vitro*, but also reduced their expression levels *in vivo*. In summary, our data suggested that empagliflozin protects the pancreatic tissues from diabetes mellitus-induced injury by downregulating the NLRP3/caspase-1/GSDMD pyroptosis-related inflammasome pathway.

## Graphical Abstract

**Figure:**
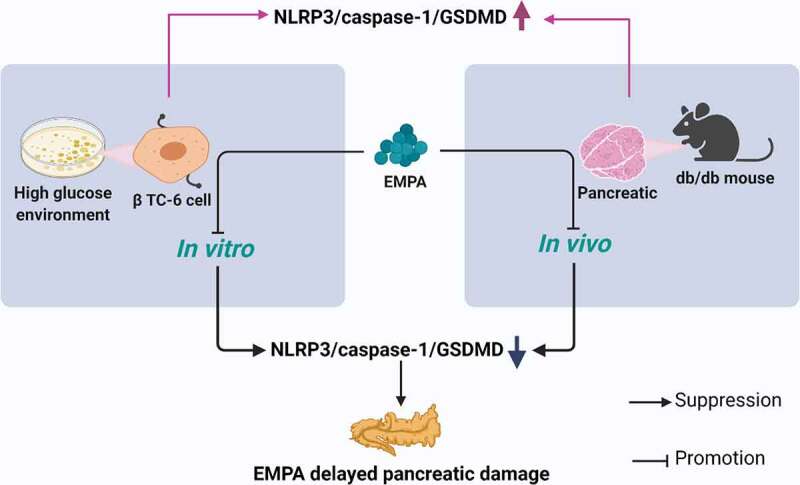

## 1. Introduction

Diabetes is a chronic metabolic disease characterized by an increase in blood sugar levels due to the body’s resistance to insulin or its inability to produce enough insulin [1], which can lead to serious complications over time [2,3]. In 2019, a total of 463 million people worldwide were living with diabetes; this number is expected to increase to 578 million by 2030 [4]. Although the pathogenesis of type 1 and type 2 diabetes mellitus is different, pancreatic β cell dysfunction and death are critical for the development of type 1 and type 2 diabetes mellitus [5–9]. Thus, understanding the mechanisms of pancreatic β cell dysfunction is critical for preventing or reversing these diseases.

Pyroptosis is a recently identified type of programmed cell death [10,11]. It is a form of inflammation activated by pathogens, such as bacteria, or their endotoxins. When the nucleotide-binding oligomerization domain-like receptor protein 3 (NLRP3) inflammasome is activated, it further activates caspase-1, which then cleaves Gasdermin D (GSDMD) and releases its N-terminal domain, thus, perforating the cell membrane and forming a membrane pore. Membrane rupture, cell osmolysis, DNA lysis, and the release of cell contents and inflammatory mediators, such as interleukin (IL)-1β and IL-18, leads to a strong inflammatory response [12–14]. Studies have shown that the expression of IL-1β is significantly increased in islet tissues after exposure to high glucose concentrations, and IL-1β increases the toxicity of the islet tissues.

In one study, NLRP3^(-/-)^ mice had improved glucose tolerance and insulin sensitivity [15]. NLRP3 promotes islet fibrosis and increases insulin resistance in mice. However, elimination of NLRP3 in the pancreatic β cells of mice protected them from cell death caused by a long-term high-fat diet and significantly increased islet volume [16]. Other studies have found that NLRP3-mediated inflammation is triggered when high glucose and lipid toxicity disturb islet function; this assists mitochondria to release oxygen free radicals and further disrupt islet function [17]. In contrast, Wali et al., through knockout experiments and overexpression of NLRP3 by pancreatic β cells induced by high glucose and lipid levels, found that there were no significant changes in the expression of IL-1β, the degree of injury, and the death rate of pancreatic β cells, suggesting that pancreatic β cells do not undergo pyroptosis [18]. In addition, Wang et al. found that NLRP3 deficiency in Akita mice did not affect the onset or severity of diabetes. Moreover, deletion of NLRP3 in Akita mice did not prevent endoplasmic reticulum stress-mediated β cell dysfunction [19]. Therefore, the involvement of pyroptosis in the damage of pancreatic β cells and progression of diabetes is still controversial.

Selective sodium-glucose cotransporter 2 inhibitors provide an insulin-independent mechanism to improve blood glucose levels and are approved for the treatment of type 2 diabetes mellitus [20]. They promote urinary glucose excretion by inhibiting (up to approximately 50%) urinary glucose reabsorption in the proximal tubules of the kidney [21,22]. Empagliflozin is the most selective drug currently available against selective sodium-glucose cotransporter 2 [23,24]. Previous studies have shown that inhibition of selective sodium-glucose cotransporter 2 reduces glucose toxicity and pancreatic β cell mortality and achieves glycemic control by protecting pancreatic β cell function and endogenous insulin secretion [25]. Empagliflozin reduced oxidative stress and cell death in the pancreatic and kidney tissues of type 2 diabetes mellitus mice [26]. Although several studies have shown that selective sodium-glucose cotransporter 2 inhibitors can improve myocardial, renal, and pancreatic pathological status in db/db mice, the effects of selective sodium-glucose cotransporter 2 inhibitors on pyroptosis of pancreatic β cells in db/db mice remains unclear.

Therefore, we hypothesized that empagliflozin may play an important role in the pancreatic tissues in db/db mice. To determine whether empagliflozin had a potential effect on the pancreatic tissues in db/db mice and pancreatic β cells induced by high glucose, we evaluated the expression of pyroptosis-related inflammatory factors in the pancreatic tissues in db/db mice and pancreatic β cells in a high-glucose environment and verified the effect of the selective sodium-glucose cotransporter 2 inhibitor, empagliflozin, on these factors.

## 2. Materials and methods

### 2.1. Reagents and instruments

Dimethyl sulfoxide (DMSO) (D8371, Solaibao, Beijing, China); Cell Counting Kit-8 (CA1210, Solaibao); BCA Protein Concentration Determination Kit (P0012S, Beyotime, Shanghai, China); RIPA buffer (R0010, Solaibao); Hematoxylin and Eosin Staining Kit (C0105S, Beyotime); 4% paraformaldehyde (BL539A, Biosharp, Hefei, China); 10% neutral buffer formalin fixative (Thermo right, Changchun, China); TMB Chromogen Solution (P0211, Beyotime); DAPI (C0065, Solaibao); Anti-GSDMD antibodies (ab219800, Abcam, Cambridge, UK); Anti-NLRP3 antibodies (ab214185, Abcam); Anti-caspase-1 antibodies (ab138483, Abcam); β-actin polyclonal antibody (20,536-1-AP, Proteintech, Rosemont, IL, USA); Anti-caspase-1 antibody (AB1871, Sigma,-Aldrich, USA); NLRP3 recombinant rabbit monoclonal antibody (sc06-23, Invitrogen, Waltham, MA, USA); Cy3-labeled goat anti-rabbit IgG (H + L) (A0516, Beyotime); Goat-anti-rabbit IgG (H + L) (A0208, Beyotime); Biotin-labeled Goat Anti-Rabbit IgG(H + L) (A0277, Beyotime); Empagliflozin (HY-15409, MedChemExpress, Princeton, NJ, USA); ECL Kit (Millipore, Billerica, MA, USA). CO_2_ constant temperature incubator (Thermo Fisher, USA); fluorescence microscope (DM500, Leica, Germany); Electrophoresis instrument electrophoresis system (Bio-Rad, Hercules, CA, USA); ChemiDoc™MP system (Bio-Rad).

### 2.2 In vitro *experiment*

#### 2.2.1 Cell culture

β TC-6 cells were purchased from Geneo Bio Co, Ltd. and cultured in complete medium containing 15% fetal bovine serum and 1% double antibody in a 5% CO_2_ environment at 37°C. The cells were grown to approximately 80% density for trypsin digestion and resuspended in a low glucose complete medium. They were then divided into three groups according to the intervention methods: A. Blank control group with low-glucose medium (Control) group: complete culture medium with glucose 1 g/L; B. High glucose group: complete culture medium with 6 g/L glucose; C. High glucose+EMPA group: cells were pretreated with 6 g/L high glucose complete medium for 2 h, and then an appropriate amount of empagliflozin was added at a concentration of 200 nmol/L in the complete medium. All the groups were processed for 24 h [27].

#### 2.2.2 Cell Counting Kit-8 (CCK-8) assay

The protocol for the cell counting kit-8 was described in a previous study [28]. β TC-6 cells were seeded into 96-well plates at a density of 2 × 10^4^ cells/well and incubated overnight, and the supernatant was discarded after stabilization and adhesion. Two interventions were used in this experiment and the concentration gradients were as follows: (A) glucose (0, 1, 2, 6, and 10 g/L), (B) empagliflozin (0, 50, 100, 200, and 500 nmol/L). Each group comprised five wells. At 6, 16, and 24 h, 10 µL CCK-8 reagent was added to each well. After incubation at 37°C for 60 min at 5% CO_2_, the absorbance was detected at 450 nm using a microplate instrument.

#### 2.2.3 Western blotting *assay*

Protein was harvested from the cells as described previously [29]. The supernatant of the treated cells was discarded, and the cells were rinsed with phosphate-buffered saline (PBS), and a 99:1 lysate and protease inhibitor (phenylmethylsulfonyl fluoride) solution was added to obtain whole cell lysates. The whole cell lysates were then transferred into 1.5 mL Eppendorf (EP) tubes, numbered, and centrifuged at 12,000 rpm at 4°C for 15 min. The supernatant was decanted and the residual samples were transferred into 0.5 mL EP tubes. After adding the loading buffer, the samples were denatured at 100°C for 10 min and placed in a – 80 ℃ refrigerator for later use. Protein concentrations were determined using the bicinchoninic acid method. The denatured proteins were isolated using a 10% sodium dodecyl sulfate polyacrylamide gel electrophoresis gel and then transferred to a polyvinylidene fluoride membrane, which was then sealed for 60 min. The cells were incubated overnight at −4°C with anti-GSDMD (ab219800; 1:500), anti-NLRP3 (ab214185; 1:500), anti-caspase-1antibodies (ab138483; 1:500), and β-actin polyclonal antibody (20,536-1-AP, 1:2000). After washing with tris-buffered saline with Tween for 10 min, the cells were incubated with Biotin-labeled Goat Anti-Rabbit IgG (H + L) (A0277, 1:2000) for 2 h in a shaker room at room temperature. Protein expression levels were tested using an ECL kit, imaged on a Bio-Rad ChemiDoc™MP system, and analyzed using Image-Pro-Plus 6.0.

#### 2.2.4 Cellular immunofluorescence

Immunofluorescence of the pancreatic β cells was performed as previously described [30]. Briefly, pancreatic β cells (2 × 10^4^) were cultured in 24-well culture plates, fixed with 4% paraformaldehyde for 30 min, washed twice with PBS, and then permeabilized with methanol for 10 min at room temperature. The cells were then stained with anti-caspase-1 antibody (AB1871; 1:200) and NLRP3 recombinant rabbit monoclonal antibody (SC06-23; 1:200) at 4°C overnight, and subsequently Cy3-labeled goat anti-rabbit IgG (H + L) (A0516; 1:200) was used in the dark at 37°C for 1 h. Nuclei were stained with DAPI for 10 min. Images were captured using a fluorescence microscope (20 × 10 magnification). Data were analyzed using the Image-Pro-Plus 6.0 software.

### 2.3 In vivo *experiment*

#### 2.3.1 Animal models

Forty-seven eight-week-old male BKS-Lepr^em2Cd479^/Gpt mice and 15 eight-week-old male C57/bl6j mice were purchased from Jiangsu Jiangsu Jicui Yaokang Biotechnology Co. (Jiangsu, China). All mice were raised at the Laboratory Animal Experiment Center of Chengdu Medical College. The research protocol was reviewed and approved by the Animal Research Committee of Chengdu Medical College. The experiments were carried out in accordance with the Chinese National Standard ‘Experimental Animals and Environmental Facilities’ and the experimental animal experiment regulations of Chengdu Medical College on animal care and use.

Fifteen C57/bl6j wild-type mice were designated as the blank control group (control group), and fed with ordinary feed and distilled water. Twenty-five BKS-Lepr^em2Cd479^/Gpt mice were assigned as diabetes models (db/db group) [31], and were also fed with ordinary feed and distilled water. Twenty-two BKS-Lepr^em2Cd479^/Gpt mice were set as the empagliflozin group (db/db+EMPA group), and they were administered empagliflozin at 10 mg/(kg·day) by gavage. The clean tablets were ground into powder and dissolved in double-distilled water, calculated at 10 mg/kg per mouse, supplemented with ordinary feed and distilled water. After feeding the mice in each group for six months according to the above grouping and feeding methods, mice were sacrificed by cervical dislocation under anesthesia with sodium pentobarbital (1%, 50 mg/kg). After the mice were immersed and disinfected in alcohol, their pancreas were surgically removed and numbered, and the pancreatic tissues were placed into a sealed tube containing 10% neutral specimen fixative and stored in a refrigerator at 4°C for later use.

#### 2.3.2 Hematoxylin-eosin staining

The procedure of hematoxylin-eosin staining is similar to that of a previous study [32]. The tissue sections of the pancreas were deparaffinized in xylene, dehydrated with ethanol, stained with hematoxylin staining solution, and differentiated with hydrochloric acid and ethanol slow differentiation solution; eosin was then used for counterstaining. Ethanol was used for gradient dehydration again, xylene was used for transparency, and images were captured using a fluorescence microscope (20 × 10). Data were analyzed using the Image-Pro-Plus 6.0 software.

#### 2.3.3 Immunohistochemistry

Immunofluorescence of the pancreatic tissues was performed as previously described [33]. Fixed pancreatic tissues were made into paraffin sections, which were placed in a slice rack, and melted on slides in a 60°C oven. After that, the slices were baked and placed in a xylene glass jar for deparaffinization and rehydration; 0.01 mol/L citric acid-sodium citrate buffer was used to steam for 30 min to perform antigen retrieval. Hydrogen peroxide (3%) was used for the inactivation. According to the manufacturer’s instructions, the recombinant anti-GSDMD, anti-NLRP3, and anti-caspase-1 antibodies were diluted with PBS at a ratio of 1:500, then dropped onto a glass slide for incubation with primary antibody, and then placed in the refrigerator at 4°C overnight. According to the instructions, horseradish peroxidase-labeled goat-anti-rabbit IgG (H + L) was diluted with PBS in a ratio of 1:50 for incubation of secondary antibody, and then placed in an incubator at 37°C for 30 min. The TMB Chromogen Solution was added and incubated in the dark for 60 min. Finally, images were captured using a fluorescence microscope (20 × 10). Data were analyzed using the Image-Pro-Plus 6.0 software.

### 2.4 Statistics analysis

Statistical analyses were performed using GraphPad Prism 8.0.2. Data are expressed as the mean ± standard deviation (mean ± SD), and the results of the CCK-8 assay were statistically calculated using a two-factor analysis of variance. The remaining results were compared between the groups using one-way analysis of variance (ANOVA) and post hoc Tukey’s test. Statistical significance was set at *P*< 0.05.

## 3 Results

We hypothesized that empagliflozin may play an important role in the pancreatic tissues in db/db mice. In this study, we intervened β TC-6 cells to detect the effects of high glucose and empagliflozin on pyroptosis-related NLRP3, caspase-1, and GSDMD in β TC-6 cells. In addition, we also observed the effects of empagliflozin on NLRP3, caspase-,1 and GSDMD in pancreatic tissues of db/db mice. We found that empagliflozin not only reduces the expression of these factors in β TC-6 cells in a high glucose environment *in vitro*, but also reduced their expression in the pancreatic tissue of db/db mice *in vivo* and protected the pancreas from damage.

### 3.1 Effects of glucose and empagliflozin on β TC-6 cell proliferation as determined by the Cell Counting Kit-8 assay

**Figure 1.**
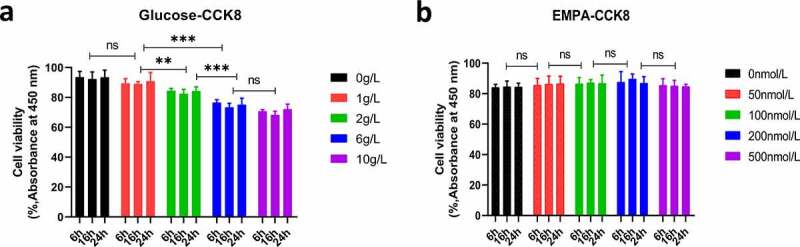
Cell viability was determined by using the Cell Counting Kit-8 (CCK8) viability assay. (a) The β TC-6 cell viability was approximately 75% when the glucose concentration was 6 g/L, and the inhibitory effect was strongest when the intervention lasted 16 h. (b) The concentration of EMPA was 200 nmol/L, and the β TC-6 cell activity was highest when the continuous intervention for 16 h. ns *P* > 0.05; * *P* < 0.05; ** *P* < 0.01; *** *P* < 0.001. β TC-6 cell: Mouse insulinoma islet β cells; CCK8: Cell Counting Kit-8; EMPA: Empagliflozin

The inhibitory effect of glucose on β cell activity was concentration-dependent, and the cell viability was approximately 75% when the glucose concentration was 6 g/L, and the inhibitory effect was strongest when the intervention lasted 16 h (Figure 1a). For empagliflozin, the cell activity was highest with continuous intervention for 16 h at 200 nmol/L (Figure 1b). These results provide reasonable intervention concentrations and time parameters for subsequent studies.

### 3.2 Empagliflozin can inhibit the high glucose-induced increase in the expression of NLRP3, caspase-1, GSDMD in β TC-6 cells

**Figure 2.**
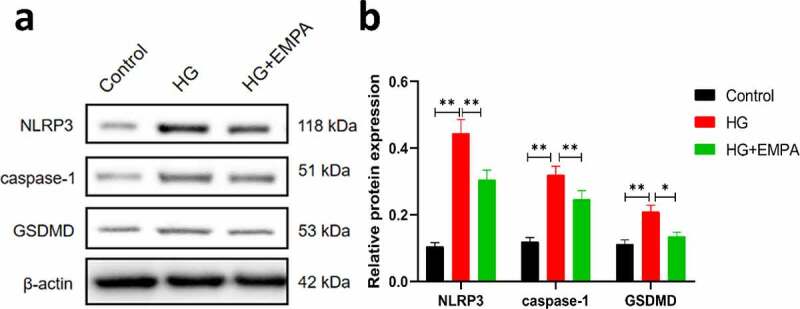
The expressions of pyroptosis-related proteins (NLRP3, caspase-1 and GSDMD) of β TC-6 cells in different environment were analyzed by western blotting. (a) The images of electrophoretic bands. (b) β TC-6 cells were under three different treatment conditions as described above for 24 h. ns *P* > 0.05; * *P* < 0.05; ** *P* < 0.01; *** *P* < 0.001. NLRP3: *nucleotide-binding oligomerization domain-like receptor protein 3*; GSDMD: *Gasdermin D.*

**Figure 3.**
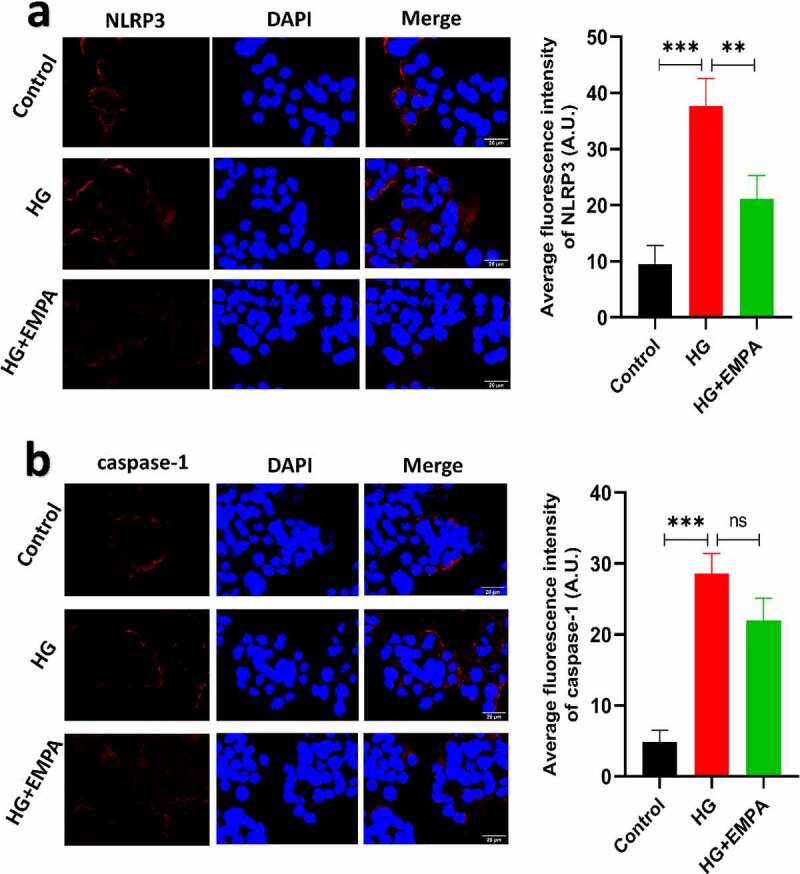
The results of immunofluorescence of β TC-6 cells treated with different conditions as described above for 24 h. (a) and (b) Immunofluorescence images showing expressions of NLRP3 and caspase-1 in β TC-6 cells under three different treatment conditions. * *P* < 0.05; ** *P* < 0.01; *** *P* < 0.001

Using *western blotting*, we assessed the expression of NLRP3, caspase-1, and GSDMD expression levels in β TC-6 cells in a high glucose environment and also the expression of these molecules in β TC-6 cells treated with empagliflozin in the high glucose environment. The results showed that the expression of NLRP3, caspase-1, and GSDMD in β TC-6 cells increased in the high glucose environment, but empagliflozin inhibited their expression (Figure 2a–b). We found similar results using the immunofluorescence assay (Figure 3a–b).

### 3.3 Empagliflozin can downregulate the expression of NLRP3, caspase-1, and GSDMD in the pancreatic tissues of db/db mice

**Figure 4.**
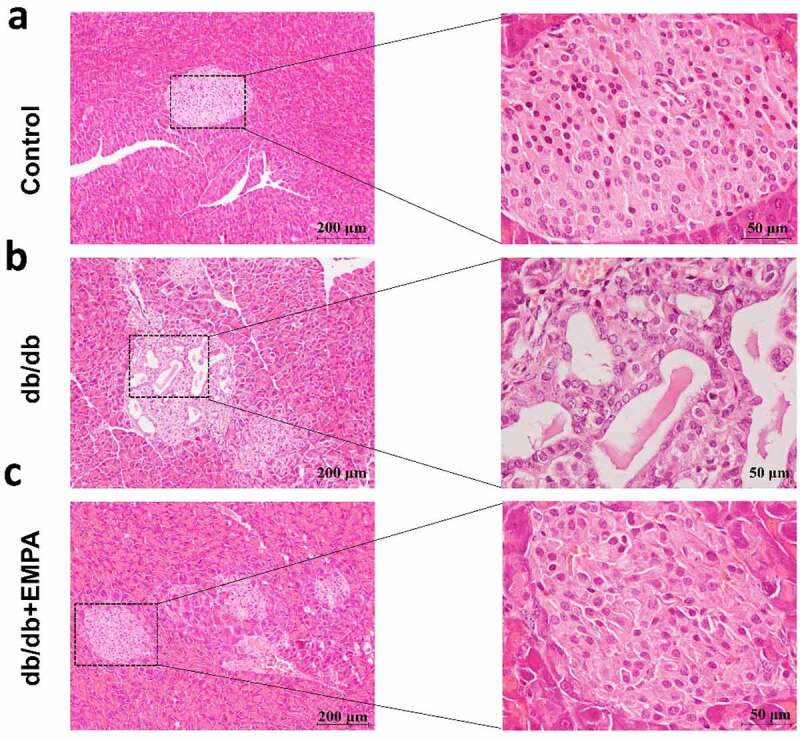
Hematoxylin and Eosin staining of histological sections of mice pancreas. (a) The blank control group. (b) The db/db group. (c) The db/db+EMPA group

**Figure 5.**
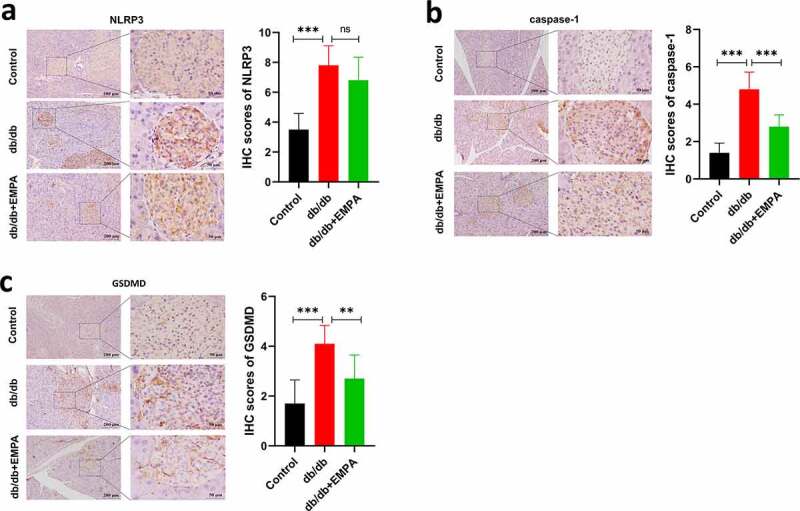
The protein expressions of NLRP3, caspase-1 and GSDMD were assumed by immunohistochemistry. (a) Immunohistochemical images and scores of NLRP3 of pancreas in mice treated with different methods. (b) Immunohistochemical images and scores of caspase-1 of pancreas in mice treated with different methods. (c) Immunohistochemical images and scores of GSDMD of pancreas in mice treated with different methods. ns *P* > 0.05; * *P* < 0.05; ** *P* < 0.01; *** *P* < 0.001. NLRP3: *nucleotide-binding oligomerization domain-like receptor protein 3*; GSDMD: *Gasdermin D.*

**Figure 6.**
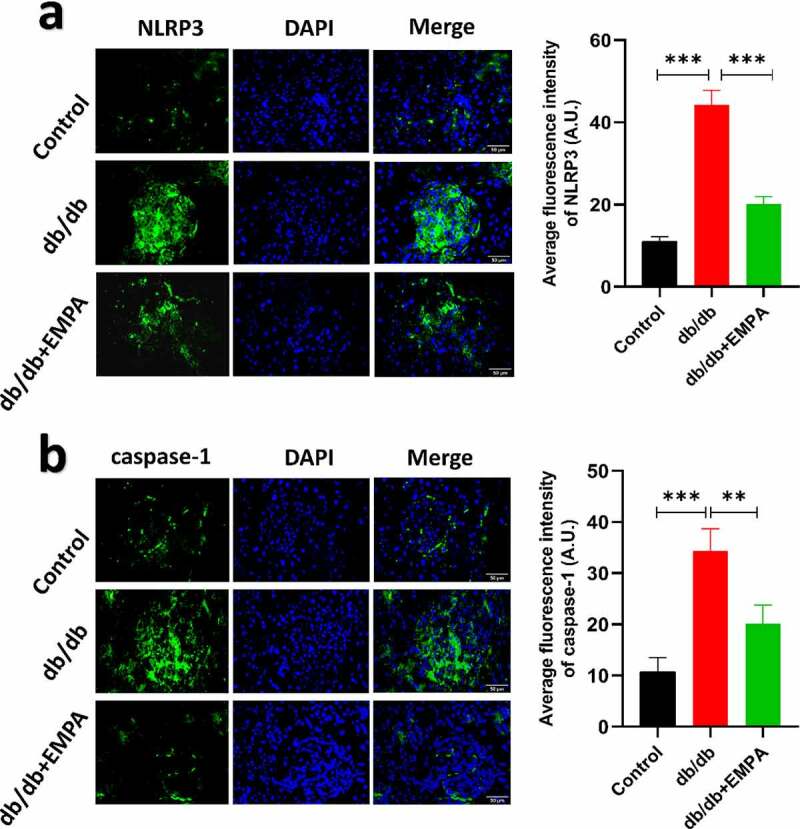
The results of immunofluorescence of in pancreas of mice treated with different methods as described above for six months. The expressions of NLRP3 (a) and caspase-1 (b) under three different treatment methods by immunofluorescence images and scores. ns *P* > 0.05; * *P* < 0.05; ** *P* < 0.01; *** *P* < 0.001. NLRP3: *nucleotide-binding oligomerization domain-like receptor protein 3.*

To further investigate the role of empagliflozin in inflammatory factors related to pyroptosis in the pancreatic tissue, we used a mouse model of diabetes. The results of hematoxylin-eosin staining showed that the islet tissue of the non-diabetic group was distributed in a round or oval shape, with complete structure, clear borders, no inflammatory cell infiltration, hemorrhage, or necrosis (Figure 4a). In the db/db mice group, the pancreatic islet tissue showed a cord-like or irregular distribution, with incomplete structure, unclear borders, disordered pancreatic islet cell arrangement, duct proliferation and expansion (eosinophilic secretions in the cavity), and pancreatic islet fibroblast proliferation. Inflammatory cell infiltration was observed in the pancreatic stroma (Figure 4b). In the db/db+EMPA group, the pancreatic islet tissue was cord-shaped, with complete structure, clear borders, neatly arranged islet cells, red staining of the islet cell cytoplasm (amyloidosis), and a small amount of islet fibroblast proliferation. Neither inflammatory cell infiltration nor hemorrhagic necrosis was observed in the pancreatic stroma (Figure 4c). Immunochemistry analysis showed that the expression of NLRP3, caspase-1, and GSDMD in the pancreatic tissue of db/db mice was increased, and empagliflozin could inhibit their expression (Figures 5a–5 c). Immunofluorescence analysis of pancreatic tissue showed similar results (Figure 6a–b). These results indicate that empagliflozin can inhibit pyroptosis-related inflammatory factors of and retard the progression of pancreatic tissue damage in db/db mice *in vivo*.

## 4 Discussion

Although a variety of drugs and surgical methods have been used to treat diabetes, the results have not been satisfactory. Many researchers are trying to delve deeper into the pathological mechanisms of the disease in order to obtain better treatment methods [34].

Previous studies have shown that NLRP3 is expressed in a variety of cells and its mediated signaling pathway plays an important role in the progression of diabetes [35,36]. Several studies have shown that activation of NLRP3 can stimulate inflammatory formation and pyroptosis of cells [37,38]. These findings prompted us to detect NLRP3 expression in db/db mice and analyze its association with pancreatic β cells.

In this study, BKS-Lepr^em2Cd479^/Gpt mice were used as db/db mice, which were leptin receptor (Lepr) gene mutant mice constructed using CRISPR/Cas9 technology and fertilized egg injection technology with C57BLKS/JNju mice as background. A number of studies have used this mouse as a model of diabetes after Lepr gene mutation caused protein dysfunction, which led to increased blood glucose and body weight [39,40]. Our results showed that NLRP3 expression was significantly increased in db/db mice compared with control group, and expression levels of caspase-1 and GSDMD were also elevated in subsequent tests. However, empagliflozin reduced NLRP3/caspase-1/GSDMD expression in the pancreatic tissues of db/db mice. In the db/db group, the pancreatic islet tissues showed a cord-like or irregular distribution, with incomplete structure, unclear borders, disordered pancreatic islet cell arrangement, duct proliferation and expansion, and pancreatic islet fibroblast proliferation. Inflammatory cell infiltration was also observed in the pancreatic stroma. In contrast, db/db mice treated with empagliflozin significantly improved these pathological changes in pancreatic tissues. In conclusion, empagliflozin has a protective effect on the pancreatic tissues, and this protective effect may be realized through theNLRP3/Caspase-1/GSDMD pathway.

Empagliflozin has a unique insulin-independent hypoglycemic pathway and can exert hypoglycemic effects even when the function of pancreatic β cells is impaired [41,42]. The *in vitro* experiment does not involve the effect of selective sodium-glucose cotransporter 2 inhibitors on the reabsorption mechanism of renal tubules, which avoids the effect of empagliflozin on the reabsorption of sugar by the renal tubules as in the *in vivo* experiments. Therefore, we designed a study on the direct effect of empagliflozin on pancreatic β cells *in vitro*. Pancreatic β cells attacked by high glucose are a common diabetic cell model since insulin deficiency caused by pancreatic β cells dysfunction is an important pathogenic factor of diabetes mellitus [27,43,44]. Consistent with previous studies [45–47], using the CCK-8 assay, we found that the higher the concentration of glucose, the lower the activity of pancreatic β cells. Subsequently, we found elevated levels of NLRP3/caspase-1/GSDMD expression in high glucose-induced pancreatic β cells, which is similar to the results of a previous study [48]. Consistent with the results of the *in vivo* studies, empagliflozin downregulated the expression level of NLRP3/caspase-1/GSDMD in pancreatic β cells *in vitro*. In regards to previous reports, it is suggested that in high glucose-induced cell models, empagliflozin can reduce the occurrence of pyroptosis-related inflammatory bodies in cardiomyocytes and kidney cells [49], as well as reduce the occurrence of pyroptosis-related inflammatory bodies in pancreatic β cells. It is well-known that insufficient pancreatic β cell function can lead to decreased insulin secretion, which further aggravates the development of diabetes.

Selective sodium-glucose cotransporter 2 inhibitors can preserve pancreatic β cell function and endogenous insulin secretion by reducing glucose toxicity and rates of pancreatic β cell death, but the specific mechanism is unclear [25,47,50,51]. Our *in vitro* study confirmed that empagliflozin reduced the activation of NLRP3 and the expression of pyroptosis-related inflammasomes in pancreatic β cells under a high glucose environment, suggesting that the NLRP3/caspase-1/GSDMD pathway may be a potential therapeutic target for diabetes.

Our research clarified the effect of empagliflozin on the pyroptosis-related inflammatory factor, NLRP3/Caspase-1/GSDMD, in β TC-6 cells in a high-glucose environment and pancreatic tissues of db/db mice, and revealed the effect of empagliflozin on pancreatic tissue damage in db/db mice. However, there are several limitations to our study. First, the effect of empagliflozin on β TC-6 cells in a high glucose environment may not be limited to pyroptosis-related proteins and pathways. The mechanisms involved may be diverse and multifaceted. Second, in addition to the NLRP3/caspase-1/GSDMD pathway, many other proteins or pathways may be involved in the protective effects of empagliflozin in the pancreatic tissue of db/db mice. Hence, further research on the role of empagliflozin in the relationship between diabetic pancreatic β cells and pyroptosis-related inflammasomes is required.

## 5 Conclusions

Empagliflozin inhibits the activation of NLRP3/caspase-1/GSDMD inflammasomes in pancreatic β cells in high glucose environments *in vitro* and reduces the expression of NLRP3, caspase-1, and GSDMD in the pancreatic tissue of db/db mice *in vivo*, thus protecting the pancreas from damage.

## Research highlights

1. Empagliflozin inhibits NLRP3/caspase-1/GSDMD inflammasome activation *in vitro.*

2. Empagliflozin reduces NLRP3, caspase-1, and GSDMD expression in the pancreas *in vivo.*

3. Empagliflozin can protect the pancreas from diabetes mellitus-induced damage.
